# Breathing exercises: influence on breathing patterns and thoracoabdominal motion in healthy subjects

**DOI:** 10.1590/bjpt-rbf.2014.0048

**Published:** 2014

**Authors:** Danielle S. R. Vieira, Liliane P. S. Mendes, Nathália S. Elmiro, Marcelo Velloso, Raquel R. Britto, Verônica F. Parreira

**Affiliations:** 1 Curso de Fisioterapia, Universidade Federal de Santa Catarina (UFSC), Araranguá, SC, Brazil; 2 Programa de Pós-graduação em Ciências da Reabilitação, Departamento de Fisioterapia, Universidade Federal de Minas Gerais (UFMG), Belo Horizonte, MG, Brazil; 3 Programa de Residência Multiprofissional, Hospital das Clínicas, UFMG, Belo Horizonte, MG, Brazil; 4 Departamento de Fisioterapia, UFMG, Belo Horizonte, MG, Brazil

## Abstract

**BACKGROUND:**

The mechanisms underlying breathing exercises have not been fully elucidated.

**OBJECTIVES:**

To evaluate the impact of four on breathing exercises (diaphragmatic breathing, inspiratory sighs, sustained maximal inspiration and intercostal exercise) the on breathing pattern and thoracoabdominal motion in healthy subjects.

**METHOD:**

Fifteen subjects of both sexes, aged 23±1.5 years old and with normal pulmonary function tests, participated in the study. The subjects were evaluated using the optoelectronic plethysmography system in a supine position with a trunk inclination of 45° during quiet breathing and the breathing exercises. The order of the breathing exercises was randomized. Statistical analysis was performed by the Friedman test and an ANOVA for repeated measures with one factor (breathing exercises), followed by preplanned contrasts and Bonferroni correction. A p<0.005 value was considered significant.

**RESULTS:**

All breathing exercises significantly increased the tidal volume of the chest wall (V_cw_) and reduced the respiratory rate (*RR*) in comparison to quiet breathing. The diaphragmatic breathing exercise was responsible for the lowest V_cw_, the lowest contribution of the rib cage, and the highest contribution of the abdomen. The sustained maximal inspiration exercise promoted greater reduction in *RR* compared to the diaphragmatic and intercostal exercises. Inspiratory sighs and intercostal exercises were responsible for the highest values of minute ventilation. Thoracoabdominal asynchrony variables increased significantly during diaphragmatic breathing.

**CONCLUSIONS:**

The results showed that the breathing exercises investigated in this study produced modifications in the breathing pattern (*e.g.,* increase in tidal volume and decrease in *RR*) as well as in thoracoabdominal motion (*e.g.,* increase in abdominal contribution during diaphragmatic breathing), among others.

## Introduction

Breathing exercises are manual techniques commonly used in clinical practice. They can affect breathing patterns and thoracoabdominal movement, prioritize one compartment of the chest wall (CW) over another, and change the degree of participation of the respiratory muscles^1^.

Diaphragmatic breathing is one of the most widely used and studied exercises in clinical practice^2^^-^ 5. It aims to improve pulmonary ventilation, mainly to the dependent zones of the lungs by promoting greater respiratory displacement of the abdominal compartment^2^^-^ 4 ^,^ 6. Other breathing exercises are also used in respiratory physical therapy practice. Inspiratory sighs and sustained maximal inspiration (SMI) are used to improve lung volume and hematosis by performing successive inspirations (i.e. inspiratory sighs) or maximal inspiratory effort^7^^,^ 8. In addition, the intercostal breathing exercise emphasizes rib cage (RC) compartment muscles, promoting greater displacement of this compartment^8^^,^ 9. Cuello et al.^8^ was the first to propose the use of inspiratory sighs and intercostal breathing exercises^8^. 

The mechanisms underlying breathing exercises, particularly inspiratory sighs, SMI, and intercostal breathing, are not fully elucidated. Because the literature on these exercises is scarce, health professionals prescribe them based on the positive outcomes observed from their use or on their proposed mechanism of action. The comprehension of which CW compartments are primarily engaged during these breathing exercises may support the application of a specific exercise to conditions affecting different lung zones.

Currently, breathing pattern and thoracoabdominal movement can be assessed by optoelectronic plethysmography (OEP). This device performs a tricompartmental analysis of volume variations without presetting the degree of freedom of the CW, thus allowing a more detailed analysis of the effects of breathing exercises on the ventilation of different CW compartments (pulmonary rib cage - RCp, abdominal rib cage - RCa, and abdomen - AB)^10^^,^ 11.

The breathing exercises assessed in this study were selected based on their effect on different lung zones^1^. Because pursed lip breathing is usually associated with breathing exercises in clinical practice^12^^,^ 13, it was added to the exercises with oral expiration in this study.

Thus, the study assessed the effect of breathing exercises (diaphragmatic breathing, inspiratory sighs, SMI, and intercostal breathing) on the breathing pattern and thoracoabdominal movement of healthy participants.

## Method

### Sample

This was a cross-sectional study with 15 participants who met the following inclusion criteria: age between 20-30 years; body mass index (BMI) between 18.5 and 29.99 Kg/m^2^; no disturbances in ventilatory function as assessed by pulmonary function test^14^; absence of neuromuscular diseases; and no previous knowledge of how to perform the breathing exercises. The exclusion criteria were being unable to understand and/or perform any of the procedures.

This study was approved by the Ethics Committee (ETIC 0194.0.2036000-11) of the Universidade Federal de Minas Gerais (UFMG), Belo Horizonte, MG, Brazil. All participants signed an informed consent form.

### Measurement instruments

The OEP (BTS Bioengineering, Milan, Italy) was used to assess the breathing pattern and the thoracoabdominal movement. It is a non-invasive device^15^^,^ 16 that provides an accurate and precise indirect measurement of the absolute volumes of the three compartments of the CW during both quiet breathing and exercise^17^^,^ 18 and in different positions^15^^,^ 16 ^,^ 19. Detailed information about the OEP system was recently published by our group. To acquire the measures, it is necessary to define the anatomical boundaries of the three compartments: the xiphoid process between the RCp and the RCa, and the costal margin anteriorly and the lowest point of the lower costal margin posteriorly between the RCa and the AB^20^. The device tracks the three-dimensional position and displacement of each point of the CW, acquired by six synchronized cameras that capture the light from passive markers (plastic spheres coated with reflective paper). In an orthostatic or seated position, the markers are distributed across 89 points, and for the supine position, the markers are distributed across 52 points, marking the anatomical references of the rib cage (RC) and of the AB^10^^,^ 11 ^,^ 20.

### Procedures

The data were acquired on two days with a maximum interval of one week between data collection days. On the first day, the participants were given instructions about the study; they signed the informed consent form and answered a questionnaire to collect clinical and demographic data. Then, after the measurement of body weight and height (by a calibrated scale), blood pressure (BP), respiratory rate (RR), heart rate (HR), peripheral hemoglobin oxygen saturation (SpO_2_) and perceived exertion by modified Borg scale the participants were taught how to perform the pulmonary function test (Vitalograph 2120, Buckinghan, England). After the spirometry test, the participants answered the Human Activity Profile (HAP) questionnaire^21^. Both the spirometry and the HAP were administered by the same examiner.

Next, the participants learned the following breathing exercises which were randomized (computer program): diaphragmatic breathing - the participants had to perform a slow and deep nasal inspiration emphasizing the anterior displacement of the abdomen and avoiding RC displacement^4^^,^ 22; inspiratory sighs - short, successive, and slow nasal inspirations until the inspiratory capacity was reached^7^; sustained maximal inspiration - a slow nasal maximal inspiratory effort to reach the inspiratory capacity, followed by a 3-second post-inspiratory pause^23^; and intercostal breathing - nasal inspiration emphasizing the displacement of the upper portion of the thorax^9^. For the diaphragmatic breathing, inspiratory sighs, and maximal inspiration, the subjects were instructed to perform a smooth and controlled pursed lip expiration. For the intercostal breathing, the subjects were instructed to perform the expiration nasally, as recommended in the literature^7^. 

On the second test day, the examiner placed the 52 markers at the pre-determined anatomical references on the anterior thoracoabdominal wall, and the OEP was statically and dynamically calibrated according to the established protocol^20^. 

All participants were assessed in a supine position with a 45º trunk inclination, which is the standard position used for breathing exercises in hospitals. Initially, 5 minutes of quiet breathing were recorded, defined by the participant's own breathing pattern, followed by 5 minutes of one breathing exercise. The participants performed two sessions of 2 minutes for each breathing exercise, with a 1-minute interval between them, and only the second session was considered for data analysis.

The order of the exercises was randomized, and the participants received standard instruction for each exercise at the beginning and after 60 seconds of exercise. There was a 10-minute interval between different breathing exercises for the participants to reestablish their initial HR, *RR*, SpO_2_, and modified Borg scale values. All exercises were taught and monitored by the same examiner.

### Variables

The following variables were analyzed for each exercise: chest wall tidal volume (V_cw_); *RR*; minute ventilation (*V* ^•^ *E*); pulmonary rib cage percent contribution (V_RCp%_); abdominal rib cage percent contribution (V_RCa%_); abdomen percent contribution (V_AB%_); and the variables related to thoracoabdominal asynchrony: phase-angle (PhAng) and inspiratory phase ratio (PhRIB) between the RC and AB and between the RCp and RCa.

### Statistical analysis

Due the lack of sufficient data in the literature for sample size calculation, the sample size was calculated after the assessment of V_cw_, *RR*, *V* ^•^ *E*, V_RCp%_, V_RCa%_ and V_ab%_ in 10 participants. The effect size was calculated by the square root of the sum of the squared factors, divided by the sum of the squared errors. The data were acquired from an ANOVA table generated by the software SPSS v 13.0 (Chicago, IL, USA). The sample size was calculated considering the effect size for each variable, with a significance level of 5% and 80% power^24^. Thus, the sample size was 10 participants for the variables considered for the calculation.

The data were presented as measures of central tendency and dispersion, and the normality was verified by the Shapiro-Wilk test.

Normally distributed data were analyzed using ANOVA for repeated measures with one factor (breathing exercises), followed by pre-planned contrasts and Bonferroni correction for *p* value adjustment according to the number of comparisons (n=10). Non-normally distributed data were analyzed using the analogous nonparametric test, the Friedman test. After adjustment, *p* was set to <0.005.

## Results

Of the 20 initially selected participants, 5 were excluded (3 presented with ventilatory disturbances after the pulmonary function test; 1 presented with BMI over 29.99 Kg/m^2^; and 1 did not attend the second day of data collection). Thus, fifteen participants completed the study, and the sample analyzed provided a sample comfort level of 50% relative to the ideal sample size calculated.

The participants' demographic and anthropometric data, spirometry values, and physical activity level are presented in Table 1. All participants exhibited normal pulmonary function and were considered physically active according to the HAP questionaire.

**Figure 1:**
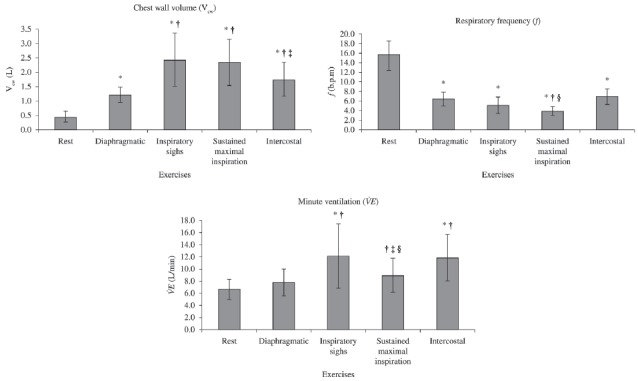
Data regarding breathing pattern variables at rest and during the four breathing exercises. Data are presented as the mean (X) and standard deviation. V_cw_: chest wall volume; *f*: respiratory frequency;*V*• *E*: minute ventilation. * p<0.005 for rest × breathing exercises; † p<0.005 for diaphragmatic breathing × inspiratory sighs, SMI and intercostal exercise; ‡ p<0.005 for inspiratory sighs × SMI and intercostal exercise; § p<0.005 for SMI × intercostal exercise.

Figure 1 shows the results of the breathing pattern variables ( i.e.V_cw_, *RR*, and* V* ^•^ *E*) at rest and during breathing exercises associated with pursed lip expiration, except for intercostal breathing (nasal expiration). V_cw_ increased significantly during all exercises relative to the rest phase. V_cw_ was significantly higher during the inspiratory sighs, SMI, and intercostal breathing compared to diaphragmatic breathing. Additionally, V_cw_ was significantly lower during intercostal breathing than during inspiratory sighs.

**Table 1:** Demographic, anthropometric, and spirometric data of the 15 subjects evaluated.

**VAR** **IÁVEIS**	X**(DP)**
Sexo	8H/7M
Idade (anos)	23,13 (1,46)
IMC (Kg/m^2^)	23,22 (2,76)
VEF_1_ (L)	3,76 (0,56)
VEF_1_ (% previsto)	94,65 (8,02)
CVF (% previsto)	92,81 (6,81)
VEF_1_/CVF	0,87 (0,05)
PAH	86,67 (5,22)

Data presented as the mean (X) with the standard deviation (SD) in parentheses. M: male; F: female; BMI: body mass index; FEV1: forced expiratory volume in one second; FVC: forced vital capacity; FEV1/FVC: ratio of forced expiratory volume in one second to forced vital capacity or *Tiffeneau *index; HAP: human activity profile.

All participants presented a significant decrease in *RR* during all exercises compared to the rest phase. The SMI caused the most significant decrease compared to diaphragmatic and intercostal breathing.

There was a significant increase in* V* ^•^ *E* during inspiratory sighs and intercostal breathing compared to the rest phase. *V* ^•^ *E* was significantly greater during inspiratory sighs, SMI, and intercostal breathing compared to diaphragmatic breathing. In addition,* V* ^•^ *E* was significantly greater during inspiratory sighs and intercostal breathing compared to SMI.

**Figure 2:**
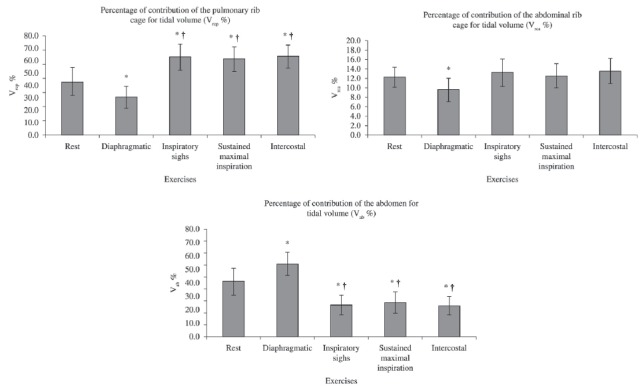
Data regarding the percentage contribution of each compartment of the chest wall (i.e. pulmonary rib cage - RCp, abdominal rib cage - RCa and abdomen - AB). Data are presented as the mean (X) and standard deviation. V_rcp_%: percentage of contribution of the pulmonary rib cage to tidal volume; V_rca_%: percentage of contribution of the abdominal rib cage to tidal volume V_ab_%: percentage of contribution of the abdomen to tidal volume. * p<0.005 for rest × breathing exercises; † p<0.005 for diaphragmatic breathing × inspiratory sighs, SMI, and intercostal exercise.

Figure 2 shows the percent contribution of each CW compartment at rest and during the breathing exercise with pursed lip expiration (except for intercostal breathing). V_RCp%_ was significantly lower during diaphragmatic breathing and significantly higher during the other exercises compared to the rest phase. Comparing the exercises revealed that V_RCp%_ was higher during inspiratory sighs, SMI, and intercostal breathing than during diaphragmatic breathing. The V_RCa%_ was significantly lower during diaphragmatic breathing than during the rest phase.

The V_ab%_ was significantly higher during diaphragmatic breathing and lower during the other exercises compared to the rest phase. V_ab%_ was significantly lower during inspiratory sighs, SMI, and intercostal breathing than during diaphragmatic breathing.

**Figure 3:**
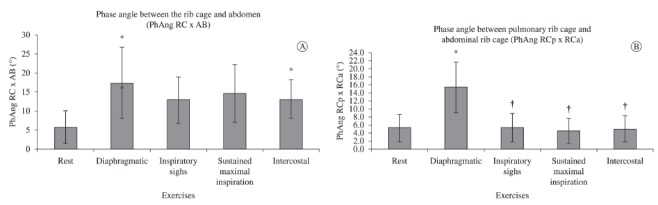
Phase angle (PhAng) between the rib cage and abdomen compartment (A) and between the pulmonary rib cage and abdominal rib cage (B). Data are presented as the mean (X) and standard deviation. RC: rib cage; AB: abdomen; RCp: pulmonary rib cage; RCa: abdominal rib cage. * p<0.005 for rest × breathing exercises; † p<0.005 for diaphragmatic breathing × inspiratory sighs, SMI, and intercostal exercise.

**Figure 4:**
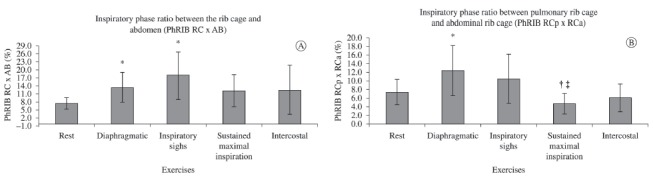
Inspiratory phase ratio (PhRIB) between the rib cage and abdomen compartments (A) and between the pulmonary rib cage and abdominal rib cage (B). Data are presented as the mean (X) and standard deviation. RC: rib cage; AB: abdomen; RCp: pulmonary rib cage; RCa: abdominal rib cage. * p<0.005 for rest × breathing exercises; † p<0.005 for diaphragmatic breathing × inspiratory sighs, SMI, and intercostal exercise; ‡ p<0.005 for inspiratory sighs × SMI and intercostal exercise.

Figures 3 and 4 show the results of the variables related to thoracoabdominal asynchrony at rest and during the breathing exercises. The PhAng results are presented in Figure 3 and show an increase between RC and AB only during intercostal and diaphragmatic breathing compared with the rest phase and no differences among the exercises. The PhAng between RCp and RCa increased significantly only during diaphragmatic breathing compared to the rest phase. Comparing the exercises showed that PhAng between RCp and RCa was significantly lower during inspiratory sighs, SMI, and intercostal breathing than during diaphragmatic breathing.

Figure 4 shows the PhRIB results. There was a significant increase in PhRIB between the RC and AB during diaphragmatic breathing and inspiratory sighs compared to rest. There was no difference among the exercises. There was a significant increase in PhRIB between the RCa and RCp during diaphragmatic breathing alone compared to the rest phase. PhRIB was significantly lower during SMI than during diaphragmatic breathing and inspiratory sighs.

## Discussion

The main findings of the study were as follows: 1) the four breathing exercises associated with pursed lip expiration and intercostal breathing, during which expiration was nasal increased V_cw_ and reduced *RR* compared to the rest phase; 2) diaphragmatic breathing increased the AB contribution significantly compared to rest and the other exercises; 3) inspiratory sighs and intercostal breathing significantly increased* V* ^•^ *E* compared to the other exercises; and 4) the thoracoabdominal asynchrony variables increased significantly during diaphragmatic breathing.

Considering the physiology of the slow and deep inspiration associated with pursed lip expiration, it is possible that increased V_cw_ associated with a reduced RR during breathing exercises contributed to a better ventilation/perfusion ratio^1^^,^ 3 ^,^ 13. 

The association between breathing exercises and pursed lip expiration reduces the *RR* because of the increased expiratory phase. The slow and extended expiration with resistance to the air outflow contributes to increase intra-bronchial pressure, improving oxygenation^7^^,^ 12 ^,^ 13. 

Diaphragmatic breathing improves pulmonary ventilation, mainly to the basal segments of the lung^2^^-^ 4. An increase in the displacement of the abdominal compartment compared to rest (with 60% contribution to V_cw_) was observed during this exercise. Therefore, this exercise can improve the contribution of the AB to the V_cw_, possibly contributing to air distribution to the basal segments of the lungs^3^. 

Other authors investigated the breathing pattern during diaphragmatic breathing in healthy subjects^2^^,^ 3 ^,^ 22. None of them, however, performed a tri-compartmental analysis of the CW, which can only be achieved using OEP.

The results of this study agree with the observations of Brach et al.^3^, who assessed the distribution of ventilation during diaphragmatic breathing and concluded that this exercise drove the ventilation from the upper segments to the lower segments of the lungs in healthy subjects without significant change in *V* ^•^ *E*. However, Tomich et al.^22^ observed a significant increase in *V* ^•^ *E* during the same exercise. The divergence between the studies could be related to the higher tidal volume associated with a small reduction in *RR* observed by these authors.

The significant increase in V_cw_ during inspiratory sighs, SMI, and intercostal breathing compared with diaphragmatic breathing may be related to performing inspiration until reaching total lung capacity, according to the proposed exercises. The higher reduction in *RR* during SMI than during diaphragmatic and intercostal breathing may be explained by the 3-second post-inspiratory pause. The absence of significant differences during the inspiratory sighs may be related to the fractionated inspirations practiced during this exercise. Although V_cw_ increased significantly during SMI, there was no significant difference in *V* ^•^ *E* compared to the rest phase. In contrast, the increase in V_cw_ during inspiratory sighs and intercostal breathing was able to compensated for *RR* reduction and promoted an increase in *V* ^•^ *E*. Inspiration performed in a single effort or in successive inspirations resulted in similar V_cw_ and reduced *RR*, but successive inspirations increased *V* ^•^ *E*. Therefore, in the presence of globally reduced pulmonary ventilation, it may be better to use this exercise instead of diaphragmatic breathing.

The execution of SMI and inspiratory sighs do not require directing airflow to a specific compartment of the CW. Furthermore, the subjects were asked to reach total lung capacity during these exercises, which might explain the small contribution of the abdominal compartment observed.

According to Fixley et al.^9^, intercostal breathing improves ventilation to the non-dependent zones of the lungs, which may be explained by the regional transpulmonary pressure gradient caused by the contraction of the CW muscles stimulated by intercostal breathing. The participants of this study were instructed to direct the airflow to the upper zone of the RC during this exercise, improving V_RCp_% and reducing V_AB_% compared to the rest phase. This finding, however, does not support nasal expiration as the factor responsible for the greater contribution of the RC to V_cw_, as proposed by the author of the technique, Cuello et al.^8^, because the effects of inspiratory sighs and SMI are similar to the effects of intercostal breathing.

PhAng is an index used to assess thoracoabdominal asynchrony^22^^,^ 25 ^-^ 27. It encompasses the data from the full respiratory cycle but assumes in its calculation that the curves formed by the movement of both compartments are approximately sinusoidal. Thus, non-sinusoidal waves can compromise its calculation. To our knowledge, only one study has assessed PhAng between RC and AB in healthy subjects, but they were assessed in a supine position with 30° trunk inclination^22^. Additionally, the measurement instrument used in that study was different from the current instrument: The OEP was used in this study, while respiratory inductive plethysmography was used in the work of Tomich et al.^22^, which does not allow a comparison between the findings. The values of the PhAng between the RCp and RCa observed in this study corroborate the findings of Aliverti et al., who assessed healthy subjects in the seated position using the OEP^27^. 

The present study showed an increase in PhAng between RC and AB during diaphragmatic and intercostal breathing exercises compared to the rest phase and showed an increase in PhAng between RCp and RCa only during diaphragmatic breathing compared to both rest and other exercises. Tomich et al.^22^ observed a significant increase in PhAng between RC and AB during diaphragmatic breathing compared to the rest phase. It is noteworthy that the thoracoabdominal asynchrony occurred mainly during exercises that required voluntary use of specific muscle groups, such as diaphragmatic and intercostal breathing, which could compromise synchrony among the compartments.

PhRIB is used to quantify asynchronous movement without the need of sinusoidal curves^28^. The values found for PhRIB agree with the values described in the literature for healthy subjects at rest^27^. The PhRIB between RC and AB showed a significant increase during diaphragmatic breathing and inspiratory sighs compared to the rest phase. There was also a significant increase in PhRIB between RCp and RCa during diaphragmatic breathing. No studies were found in the literature that assessed PhRIB during breathing exercises.

The present study showed a consistent increase in the variables related to thoracoabdominal asynchrony only during diaphragmatic breathing, both for PhRIB and PhAng. This increase is noteworthy in healthy subjects as it could be even higher in patients with chronic obstructive pulmonary diseases who present biomechanical changes of the RC. Gosselink et al.^6^ observed that patients with chronic obstructive pulmonary disease exhibited a change in the AB/RC excursion ratio during the exercises performed with or without linear load^6^. 

The results presented in the present study support the specific use of breathing exercises. The behavior of the variables analyzed in this study, even though the results were obtained from healthy participants, could be similar to the behavior of the same variables in postoperative patients because many such patients presented with previous normal pulmonary function despite all the changes occurring in this period. Therefore, the observed effects of the exercises, mainly the ones related to tidal volume increase and *RR* decrease, could benefit patients with tidal volume reduction for different reasons, such as pain, collapse of the lung parenchyma, or any other restriction. Finally, directing the ventilation to certain lung segments could improve ventilation to specific zones of the lungs of patients suffering from diseases such as atelectasis.

The indirect measurement of volume without association with a direct measure by pneumotachography is a limitation of this study. Therefore, the values recorded here cannot be used as absolute values.

## Conclusion

The findings of this study suggest that the four breathing exercises analyzed improved tidal volume and reduced *RR*. Only the diaphragmatic breathing primarily directed the ventilation to the abdominal region. Inspiratory sighs and intercostal breathing increased *V* ^•^ *E* significantly compared to the other exercises. There was no asynchrony during SMI. The results of this study may contribute to explaining the effects of these four breathing exercises on the breathing pattern and thoracoabdominal asynchrony of healthy subjects, thus allowing their proper use in clinical practice.
